# Heavy Study Investment in Italian College Students. An Analysis of Loscalzo and Giannini’s (2017) Studyholism Comprehensive Model

**DOI:** 10.3389/fpsyt.2019.00489

**Published:** 2019-07-16

**Authors:** Yura Loscalzo, Marco Giannini

**Affiliations:** Department of Health Sciences, School of Psychology, University of Florence, Florence, Italy

**Keywords:** grade point average, heavy work investment, obsession, perfectionism, study addiction, study engagement, work addiction, workaholism

## Abstract

Loscalzo and Giannini (2017) recently proposed the construct of studyholism (or obsession toward study) and a theoretical model highlighting its potential antecedents and outcomes. This study aims to analyze some of these antecedents and outcomes by means of a path analysis including both studyholism and study engagement. The participants are 1,958 Italian college students aged between 18 and 60 years (*M* age = 23.53 ± 4.43) and heterogeneous as far as their year and major of study are concerned, as well as concerning the city in which they attended their courses. They filled some instruments that allow evaluating studyholism and study engagement, along with individual and situational antecedents (e.g., worry and overstudy climate) and outcomes (e.g., sleep quality, study–relationships conflict, dropout intention). In addition to the path model we performed aiming to test the direct effects we hypothesized, we performed two MANOVAs for analyzing if there were differences on the antecedents and outcomes among the four kinds of student suggested by Loscalzo and Giannini (2017; i.e., engaged studyholics, disengaged studyholics, engaged students, and detached students). The results of this study support Loscalzo and Giannini’s (2017) conceptualization of studyholism as an internalizing disorder, since worry is the strongest predictor of studyholism (β = .67, *p* < .001). In addition, in line with Loscalzo and Giannini’s (2017) theorization, we found some differences among the four kinds of student on both the antecedents and outcomes we analyzed. This study has critical theoretical, preventive, and clinical implications. It supports the definition of studyholism as an OCD-related disorder. Also, about preventive implications, it shows that interventions aiming to favor students’ wellbeing should target also engaged students, since study engagement predicts social impairment as well as studyholism. Finally, it suggests that in a clinical setting, it is important to distinguish between disengaged studyholics and engaged studyholics as they have different relationships with some antecedents and outcomes; also, they both have functional impairment, even if in different areas.

## Introduction

Workaholism, or work addiction, is a clinical condition that has been extensively studied since its first definition at the beginning of the 1970s (1). Probably, workaholism has not yet been formally recognized in the Diagnostic and Statistical Manual of Mental Disorders, 5th edition (DSM-5) (2), despite the many peer-review papers, because it still lacks a shared definition and operationalization of criteria, which has prevented the gathering of sufficient evidence for inclusion in the manual (3–5). Moreover, almost all the papers are focused on organizational psychology and do not take into account a clinical perspective. In line with this, the first proposal of DSM-like criteria was done in 2017 by Loscalzo and Giannini (5). Loscalzo and Giannini (5) thoroughly analyzed the literature and previous workaholism models in order to propose a comprehensive theoretical model that encompasses all the main components of workaholism and is easy to test, as well as an instrument for evaluating workaholism referring to their conceptualization (i.e., Work-related Inventory, WI-10; 6). It is interesting to note that the study of Spagnoli et al. (7) supported the importance of distinguishing between engaged and disengaged workaholics, as suggested by Loscalzo and Giannini (5).

Despite workaholism being studied for almost 50 years, the analysis of a similar problem behavior in students is recent. Some studies used workaholism instruments on student samples [e.g., Refs. (8, 9)]. However, the first study that analyzed problematic overstudying with an instrument specifically developed for its assessment is the one by Andreassen et al. (10), which conceptualized problematic overstudying in the behavioral addiction framework. In contrast, Atroszko et al. (11) are the first to propose study addiction as a new area in the behavioral addiction field and to present the psychometric properties of the instrument previously used by Andreassen et al. (10) for its evaluation. Atroszko et al. (11) stated that study addiction could be analyzed from a work addiction perspective, given the many similarities between work and study.

Loscalzo and Giannini (12) recently proposed a different conceptualization of problematic overstudying that goes beyond the addiction model and that differs from that of Atroszko et al. (11) concerning some critical theoretical points (12–15). In sum, they introduced their definition of studyholism as an obsessive-compulsive-related disorder (OCD-related disorder) made up of two components (i.e., obsessive-compulsive symptoms and high or low study engagement), which led to the proposal of two subtypes of studyholics: engaged studyholics (students with high levels of both study-related obsessive-compulsive symptoms and study engagement) and disengaged studyholics (students with high levels of study-related obsessive-compulsive symptoms and low levels of study engagement).

More specifically, Loscalzo and Giannini (12, p. 31) defined (disengaged) studyholism as “a possible new clinical condition which is characterized by internalizing symptoms (i.e., obsessive-compulsive symptoms such constant thinking to study or inner drive to study) and by low levels of study engagement (something that also includes the inner motivation for studying).” Hence, they included the positive dimension of study engagement in their definition of studyholism. Study engagement, indeed, is positively associated with academic performance and success [e.g., Refs. (16–18)] and with wellbeing (17, 19–21). When introducing study engagement in their definition, Loscalzo and Giannini (12) made reference to Schaufeli et al. (22) definition, which has been derived from that of work engagement. Salanova et al. (23) assumed that students’ activities could be considered as work: students and workers are involved in structured and mandatory activities that are directed toward a goal. Consequently, the same three components of work engagement may be applied to study engagement: vigor, dedication, and absorption. Hence, Loscalzo and Giannini (12) included these three dimensions in their study engagement definition, but they also specified that intrinsic motivation should be considered as an additional component for the analysis of study engagement when analyzing studyholism.

The introduction of study engagement in the definition of studyholism, which is based on the workaholism literature (5, 24, 25), allows to specify that not all the students with high time and energy investment in study (or heavy study investors, HSIs) are studyholics. Studyholism and study engagement are indeed two different forms of heavy study investment.

In line with this, crossing the high/low levels of studyholism (or study-related obsessive-compulsive symptoms) and study engagement, it is possible to define four kinds of student, three of which are HSIs: disengaged studyholics, engaged studyholics, engaged students, and detached students (12). **Table 1** shows the four types of student.

**Table 1:** Loscalzo and Giannini’s (2017) four types of student.

Type of student	Studyholism (obsessive-compulsive symptoms) level	Study engagement level	Heavy study investor	Negative type of student
Detached Student	Low	Low	No	Yes
Engaged Student	Low	High	Yes	No
Engaged Studyholic	High	High	Yes	Yes
Disengaged Studyholic	High	Low	Yes	Yes

The detached student, being characterized by low levels of both studyholism/study-related obsessive-compulsive symptoms and study engagement, is not an HSI. This is a negative type of student, as he/she is not studyholic, but he/she is also detached from one of the most important activities in his/her life, namely, studying, which in turn could lead to negative consequences, such as low academic performance, high intention to drop out from school, and psychological impairment.

Regarding the HSIs, the engaged student is the most desirable one, characterized by low levels of studyholism/study-related obsessive-compulsive symptoms and high levels of study engagement.

Finally, the two types of studyholics are both characterized by high levels of studyholism/study-related obsessive-compulsive symptoms, but they differ in their high (engaged studyholics) or low (disengaged studyholics) levels of study engagement. Loscalzo and Giannini (12) specified that when referring to disengaged studyholics, they could simply be called studyholics, as to maintain continuity with the workaholism literature that has generally adopted a negative conceptualization. However, by introducing the engaged studyholic type, they pointed out that there could be a less impaired kind of studyholic. Engaged studyholics, even though less impaired, should receive a preventive intervention aiming to avoid their development into the disengaged type and foster instead their evolution into engaged students. In conclusion, the distinction between engaged and disengaged studyholics, as well as among the three kinds of HSI, is a core point of Loscalzo and Giannini’s (12) theorization as it allows preventing overpathologization of a common and often desirable behavior such as study (26). The authors pointed out that overstudying (or studyholism) should be considered as a pathological behavior only when it is associated with low study engagement and high impairment.

Finally, Loscalzo and Giannini (12) presented a model including both possible antecedents and outcomes of studyholism, which also distinguishes between individual and situational antecedents/outcomes. In order to develop this model, they referred mainly to Loscalzo and Giannini’s (5) workaholism model. The literature about problematic overstudying is scant (10–15, 27–29), while the workaholism literature is extensive. Hence, *keeping in mind the possible differences that could exist between workaholism and studyholism*, the knowledge about workaholism could be useful for the proposal of a studyholism model that needs to be tested in order to be adjusted based on the specific findings gathered with regard to studyholism.

More specifically, among individual antecedents, Loscalzo and Giannini (12) listed personality traits, perfectionism, motivation, cognitive factors, inability to down-regulate negative emotions, and psychiatry disorders. As far as situational antecedents are concerned, they proposed the overstudy climate, which might be spread both at school and in the family. They included the area of study as an example of situational antecedent related to the overstudy climate, as they speculate that some kind of majors (e.g., medical studies) may foster studyholism more than other courses (e.g., humanities studies). Finally, concerning studyholism outcomes, they suggested low wellbeing at school (especially in non-university students), low academic performance, physical and health impairment (including psychological disorders), and family functioning problems among the individual ones, while they listed aggressive behaviors and low positive relationships in class (especially in non-university students) among the situational outcomes.

The present study aims to shed light on the internalizing (i.e., OCD-related) and/or externalizing (i.e., behavioral addiction) nature of problematic overstudying and on the antecedents and outcomes suggested by Loscalzo and Giannini (12) as being associated with studyholism. By adopting the OCD model (12, 14, 15), it follows that two antecedents deserving attention are perfectionism and worry.

Although in the literature there are many different multidimensional conceptualizations of *perfectionism*, there seems to be consensus that it can generally be represented by very high strivings and concerns (30). These two components have different associations with positive and negative outcomes. More specifically, perfectionistic concerns (PC) is associated with higher psychopathology and lower health and wellbeing (31–35). Perfectionistic strivings (PS), instead, has a positive association with positive affect, life satisfaction, and physical health (32, 36–38), even though it seems also to be a risk factor for eating disorders and low physical health (34, 39, 40). As concerns these mixed findings about PS, Stoeber (30) suggested that the presence of both high strivings and concerns leads to negative outcomes, while the presence of elevated strivings without high concerns is generally associated with healthier adjustment.

Perfectionism has been widely studied in workers, perhaps because most people have at least one life domain in which they are perfectionistic (41), and this life domain often seems to be work, also including academic work (41, 42). Some studies showed that perfectionism is associated with lower productivity and efficiency (43, 44). Moreover, for the specific relationships between PS, PC, and work-related constructs, Stoeber and Damian (45) highlighted that the studies conducted until now showed that PS is associated with higher work engagement. PC, instead, is unrelated in some studies, while in others, it has a negative relationship with work engagement. The same relationships between PS, PC, and work engagement have been found in students too (46, 47). With regard to the relationship between perfectionism and workaholism, Clark et al. (48) recently conducted a meta-analysis on 10 studies, finding a close relationship between the two constructs. Moreover, referring to five studies published about the relationship between workaholism and perfectionism including a distinction between PS and PC, they reported that both PS and PC are positively related to workaholism (in some studies in both correlation and regression analyses, while in others only in one of the two analyses). Finally, Mazzetti et al. (49), analyzing PS only, found that it has a positive correlation with workaholism only in the context of an overwork climate. In conclusion, while both PS and PC are positively related to workaholism, PS is generally related to higher work engagement, while PC is generally associated with less work engagement (50).

Besides professional work, academic work is also a life domain often characterized by perfectionism (41, 42). However, the literature about perfectionism in academic settings is not so extensive, as could be expected (51). Rice et al. (52) summed up the literature about the relationship between academic outcomes and perfectionism by stating that generally studies have found that PS is positively associated with grade point average (GPA), while the relationship between PC and GPA is instead less consistent, and when statistically significant, PC is found to be negatively associated with GPA, and the effect size is usually small. This finding seems to be consistent across different school levels, namely, middle school, high school, and college (52).

Perfectionism is an antecedent of many psychological problems, such as depression, anxiety, eating disorders, and OCD (35, 39, 53). Regarding OCD specifically, perfectionism has been suggested as a risk factor for developing OCD (54), even though for some scholars it is better conceptualized as a predisposing trait for OCD; hence, it is necessary but not sufficient for the development of OCD (55). Perfectionism is present at high levels in people with an OCD diagnosis (53, 56–59), and it is negatively related to treatment response (59, 60). It is correlated with obsessive-compulsive symptoms also in non-clinical population (55, 61). Moreover, Pinto et al. (62), in their review of the literature about perfectionism in OCD, reported that perfectionism is also present in many OCD-related disorders, such as body dysmorphic disorder, trichotillomania, skin picking, and hoarding problems. Finally, they conclude their review by stating that OCD and perfectionism are related, especially in the dimensions of doubt about actions and concern over mistake.

Another antecedent deserving attention is worry. It is a form of repetitive negative thinking (RNT) that is usually described as the core component of general anxiety disorder (GAD; 2) and recently has been proposed as a transdiagnostic process, as it is present in most psychopathologies (63–66). As an example, it has been showed that worry is a factor contributing to OCD, panic disorder, social phobia, and depression (67–71). Worry, besides being a common process across several internalizing disorders, is associated with many negative outcomes, such as higher stress, lower physical health, and sleep problems (72–77). Moreover, trait worry is related to perfectionism (78, 79) and to higher concern over mistakes (80), which may be associated with several negative outcomes. The instrument usually used to evaluate trait pathological worry is the Penn State Worry Questionnaire (PSWQ) (81), whose items are not related to specific domains but are instead content free. Given this characteristic, the PSWQ is appropriate for evaluating trait worry (82, 83), especially when the researcher aims to evaluate its role as an antecedent of other disorders than GAD.

By means of preliminary analyses on a sample of 300 Italian college students (84), we selected six antecedents (i.e., PS, PC, study-related perfectionism, trait worry, overstudy climate, and major of study) and 12 outcomes (GPA, hours spent studying daily generally and before exams, university dropout intention, positive and negative affect, general stress, sleep quality impairment, daytime sleepiness, relationship impairment due to study, family and friends’ complaints, and aggressive behaviors at university) to study in a sample of college students. On the basis of the literature, we hypothesized that while studyholism has a negative effect on academic performance, and psychological and health wellbeing, study engagement instead has a positive effect on these variables. **Table 2** shows our hypotheses concerning both the antecedents and the outcomes.

**Table 2:** Hypotheses about the antecedents and the outcomes analyzed in the study.

Variable	Hypotheses	Literature
Perfectionistic Strivings (PS) or the perfectionism component usually associated with positive outcomes	(i) any specific hypothesis for SH	Some studies found a positive association between PS and workaholism (45), while Loscalzo (84) preliminary study found that it does not predict SH on Italian students.
	(ii) Positively predicts SE	e.g., 45, 46
	(iii) Positively predicts positive emotions	e.g., 36, 38
	(iv) Positively predicts GPA	52
	(v) Positively predicts study-related perfectionism	It is based on the consideration that study-related perfectionism is a domain-specific type of perfectionism.
Perfectionistic Concerns (PC) or the perfectionism component usually associated with negative outcomes	(i) Positively predicts SH	45
	(ii) Negatively predicts SE	However, it should be noted that while some studies support this hypothesis, other studies found the absence of a relationship between PC and work engagement (45).
	(iii) Positively predicts dropout intention	It is based on the literature supporting that PC is usually associated with negative outcomes (e.g., 31, 33).
	(iv) Negatively predicts positive emotions and positively predicts negative emotions, general stress, and sleep problems	e.g., 32, 34
	(v) Positively predicts study-related perfectionism	It is based on the consideration that study-related perfectionism is a domain-specific type of perfectionism.
	(vi) Any specific hypothesis concerning the effect on GPA	The literature shows inconsistent findings about the relationship between the two variables (52)
Study-related Perfectionism	(i) Positively predicts some study-related variables: SH, SE, GPA, time spent studying, aggressive behaviors at university, family and friends’ complaints, and the conflict between study and personal relationships	It is based on the consideration that it is a perfectionism form specifically related to study.
Trait worry	(i) Positively predicts SH	Worry is a factor contributing to OCD (68), and it is a transdiagnostic process across internalizing disorders (e.g., 64, 66)
	(ii) Negatively predicts SE	SE is a positive factor, while trait worry is a feature of internalizing disorders (e.g., 64, 66)
	(iii) Positively predicts study-related perfectionism	It is based on the literature showing that worry is related to perfectionism (79, 80)
	(iv) Positively predicts negative emotions, general stress and sleep problems, and negatively predicts positive emotions	e.g., 75–77
School and Family Overstudy Climate or the students’ perception that their family and teachers expect that they overstudy	(i) Positively predicts SH	5, 12, 49
	(ii) Negatively predicts SE	It is based on the consideration that SE is a positive factor, while overstudy climate is a variable that foster SH and workaholism (5, 12, 49)
	(iii) Overstudy climate, as communicated by means of teacher overt comments about performance, positively predicts two study-related variables, namely, study-related perfectionism and aggressive behaviors at the University	It is based on the speculation that comments about the performance may influence study-related behaviors
Area of Study—Technology, Social Sciences, Humanities, Medical, Sciences	(i) Medical studies positively predict SH and Humanities studies negatively predict SH. Any other hypothesis about the effect of the area of study on SH.	12
	(ii) Any specific hypothesis about the direction of the prediction for school-related variables: SE, GPA, time spent studying (i.e., hours per day of study generally and before exams), and social relationship impairment due to study	There are no previous studies comparing the same area of study groups on these variables.
Studyholism	(i) Does not predict GPA or negatively predicts it but with a low value (i.e., less than .20)	11, 85
	(ii) Does not predict positive emotions	preliminary study conducted by Loscalzo (84)
	(iii) Positively predicts time spent studying	12, 85
	(iv) Positively predicts the intention to drop out from university	12
	(v) Positively predicts negative emotions, general stress, sleep problems, relationship impairment due to study	e.g., 5, 11, 12, 48
	(vi) Positively predicts family and friends’ complaints about study and aggressive behaviors at the university	5, 12
Study Engagement	(i) Positively predicts GPA	e.g., 16, 18, 21
	(ii) Positively predicts time spent studying	85
	(iii) Positively predicts positive emotions	e.g., 17, 19
	(iv) Negatively predicts the intention to drop out from university	e.g., 16, 17, 21
	(v) Negatively predicts negative emotions, general stress, sleep problems, aggressive behaviors at the university, family and friends’ complaints about study and relationship impairment due to study	e.g., 19–21

SH, Studyholism; SE, Study Engagement; GPA, Grade Point Average*.*

Finally, we analyzed whether there are differences as far as studyholism (and study engagement) antecedents and outcomes are concerned among the four kinds of student proposed by Loscalzo and Giannini (12): disengaged studyholics, engaged studyholics, engaged students, detached students. Loscalzo and Giannini (12), in their theoretical model, suggested that disengaged studyholics might have higher functional impairment than have engaged studyholics and that detached students may experience negative consequence anyway. Engaged students, instead, are defined as the more desirable kind of student.

It is essential to highlight that, in order to support the OCD model (12) against the addiction model (11), the result about the predictive value of worry on studyholism seems to be the most critical. We expect to find a high predictive value of worry (i.e., a β value higher than .50). The predictive value of perfectionism seems to be less critical, as previous studies about its relationship with OCD showed that this personality trait is necessary but not sufficient for the development of OCD (55).

Also, in order to confirm Loscalzo and Giannini’s (12) model more generally, we expect to find some differences in the antecedents and outcomes between disengaged studyholics and engaged studyholics. These findings would also question the Atroszko et al. (11) model, which does not foresee the distinction between two subtypes of study addicted: with a high or low level of study engagement.

In sum, even if this is the first research to adopt Loscalzo and Giannini’s (12) model for analyzing problematic overstudying, it may have important implications for both preventive and clinical purposes (i.e., developing interventions aiming to foster academic success and students’ wellbeing). Also, it may help to shed light on the internalizing and/or externalizing nature of problematic overstudying, which until now has been analyzed from the addiction model perspective only. In this context, we want to highlight that, especially in light of comorbidity issues, we cannot define this new potential clinical condition as a pure OCD-related disorder (or as a pure behavioral addiction). We believe instead that it may be better defined as a condition more similar to an OCD-related disorder than to an addiction (12–15, 86).

## Method

### Participants

We recruited a sample of 1,958 Italian college students (75.4% females, 24.6% males) aged between 18 and 60 years (*M* age = 23.53 ± 4.43).

They attended their courses in many different Italian cities, although Florence is the most represented (39.2%). Regarding major area of study, we created the following macro groups: technology (engineering, architecture, and informatics), 11.2%; social sciences (psychology, sociology, economy, law, educational studies, etc.), 31%; humanities (literature, language, art, philosophy, history, etc.), 25.9%; medical studies, 13%; science (math, physics, biology, statistics, and chemistry), 12.8%; helping professions (nursing, obstetrics, etc.), 1.1%; and para-medical studies (biotechnology, veterinary medicine, pharmacy, etc.), 5%. The proportions of students in years 1 to 5 were 16%, 20.9%, 26.7%, 14.2%, and 15.2%, respectively. Moreover, 7% of the students reported being in their sixth year. Nearly half of these participants are medical students (it is the only course that requires 6 years for getting the master degree), while the others are students in other courses. Moreover, 65% of these students declared having been rejected during school or being currently behind in their studies. Thus, we suggest that probably non-medical students and some medical students are behind in their studies and not actually in their sixth year. Unfortunately, we did not differentiate the question about being currently late with studies or having been rejected before university; hence, further distinctions cannot be made.

### Materials

#### Study-Related Perfectionism Scale (87)

The Study-Related Perfectionism Scale (SPS) is an 11-item self-report instrument that allows an evaluation of maladaptive perfectionism in the academic context by means of four scales: excessive strivings and concerns, error intolerance, inability to delegate, and group work avoidance. The participants answer by indicating how much they agree with each item by means of a five-point Likert scale ranging between 1 (*strongly disagree*) and 5 (*strongly agree*). The SPS has good fit indices for a four-factor model (GFI = .96; CFI = .97; RMSEA = .06) and good internal reliability for its total score (α = .82), which may be calculated due to the good correlation between the factors. The SPS also has good convergent validity (87).

#### Penn State Worry Questionnaire (81)

This is a 16-item self-report instrument that measures trait worry. Since it is content free, it does not evaluate worries related to specific time frames or situations. Five items need to be reversed in order to get the total score for the test. The response format is a five-point Likert scale ranging from 1 (*strongly disagree*) to 5 (*strongly agree*). The Italian version (88) shows good psychometric properties and good internal reliability (α = .85), although four reversed items are not fully satisfactory. In the present study, the α value is even higher: .89.

#### Overstudy Climate Scale (89)

The Overstudy Climate Scale (OCS) is an 18-item self-report instrument that allows evaluating parent overstudy climate (P-OSC) and teacher overstudy climate (T-OSC). As far as T-OSC is concerned, this is evaluated by means of two scales: pressure toward hard study (T-OSC-HS) and overt comments related to the students’ academic performance (T-OSC-OC). The response format is a five-point Likert scale ranging between 1 (*strongly disagree*) and 5 (*strongly agree*). The OCS has good psychometric properties. The three-factor structure showed a good fit (GFI = .93; CFI = .97; RMSEA = .04). Moreover, the three scales have good internal consistency: P-OCS, α = .90; T-OCS-HS, α = .85; and T-OCS-OC, α = .80 (89).

#### Short Almost Perfect Scale (90)

The Short Almost Perfect Scale (SAPS) is an eight-item self-report instrument that allows evaluating both PS (standards scale) and PC (discrepancy scale). It is the short form of the Almost Perfect Scale—Revised (APS-R) (91). The participants answer on a seven-point Likert scale ranging from 1 (*strongly disagree*) to 7 (*strongly agree*). We administered the Italian version of the SAPS (92), which has a better fit for the six-item and two-factor model (CFI = .95; RMSEA = .10) than has the original eight-item version (CFI = .88; RMSEA = .13). More specifically, the Italian version of the SAPS does not include items 2 (discrepancy) and 5 (standards) in the scoring. The internal reliability of the scale is .83 for standards and .68 for discrepancy. Even though the psychometric properties of the SAPS are not fully satisfactory, we decided to retain this instrument, as other perfectionism instruments are much longer than the SAPS, and we believe it was better to have a short test, given the many instruments administered to the participants in this research. Also, the Italian and US SAPS showed partial scalar invariance, indicating functional equivalence (93).

#### Studyholism Inventory (86)

It is a 10-item self-report and brief screening instrument that allows evaluating studyholism and study engagement. It was created by an initial pool of 68 item, and its final 10-item version (with two filler items, one for each scale) has good psychometric properties (85, 86). Moreover, using the cutoffs proposed by Loscalzo and Giannini (85), it is possible to distinguish between high and low studyholism and study engagement. Moreover, by crossing high/low levels of studyholism/study engagement, it is possible to identify four kinds of student: disengaged studyholics, engaged studyholics, engaged students, and detached students. Finally, the first sheet of the instrument includes some open-format questions about study habits (e.g., studying on the weekend, and hours of study per day). The participants answer by indicating how much they agree with each item by means of a five-point Likert scale ranging between 1 (*strongly disagree*) to 5 (*strongly agree*).

#### Mini Sleep Questionnaire (94)

This is a 10-item self-report instrument evaluating sleep quality and daytime sleepiness. The participants answer by means of a seven-point Likert scale ranging between 1 (*never*) and 7 (*Always*). The Italian version (95) maintained the original two-factor structure, and it has good psychometric properties. Cronbach’s *alpha* is .75 for both sleep quality and daytime sleepiness. However, one item (snoring) is not included in the scoring for the Italian version.

#### Study–Relationship Conflict Scale (96)

The Study–Relationship Conflict Scale (SRCS) is a nine-item self-report instrument that allows evaluating the following scales: quarrels at school (QS); relationship impairment (RI), and family and friends’ complaints (FFC). The response format is a five-point Likert scale ranging between 1 (*strongly disagree*) and 5 (*strongly agree*). The three-factor structure fits the data well (GFI = .98; CFI = .98; RMSEA = .04), and the three scales have good internal reliability, especially taking into account the fact that each scale is made up of three items only: QS, α = .67; RI, α = .63; and FFC, α = .64 (96).

#### Depression Anxiety Stress Scales-21 (97)

The Depression Anxiety Stress Scales-21 (DASS-21) is a 21-item self-report scale that has been derived from a longer 42-item version (DASS) (98) by selecting seven representative items for each of the three scales (i.e., depression, anxiety, and stress). The participants answer by means of a four-point Likert scale ranging between 0 (*did not apply to me at all* - *never*) and 3 (*applied to me very much, or most of the time* - *almost always*). The Italian version (99) retained both the three-factor structure and the one-factor structure, hence allowing us to refer to a total score of general stress. Moreover, the Italian version has good psychometric properties, and, as far as the internal reliability in the community sample goes, the α values are .82 (depression), .74 (anxiety), .85 (stress), and .90 (general stress).

#### Positive and Negative Affect Schedule (100)

This is a 20-item self-report instrument that assesses two emotional dimensions: positive and negative affect. Each scale is composed of 10 items. The participants respond to each item by means of a five-point Likert scale ranging from 1 (*very slightly* or *not at all*) to 5 (*extremely*). There are two versions of the Positive and Negative Affect Schedule (PANAS), which differ in the instruction only, as they can refer to positive and negative affect as a trait, or as a state. For this study, as we were interested in affectivity as a studyholism outcome, we used the state version. The Italian version (101) has good psychometric properties, and the internal consistency is .85 for state negative affect and .83 for state positive affect.

#### Intention to Drop Out of University (102)

We used the Italian translation (103) of the three items used by Hardre and Reeve (102) for evaluating the intention to drop out of school. Two items have been shown to predict dropping out one year later (104). The response format of the three items is on a five-point Likert scale ranging from 1 (*never*) to 5 (*very often*). For the present study, we changed the word “school” to “university” in two items.

### Procedure

Once we obtained ethical approval from the University of Florence in order to gather the data and to conduct the present research, we created an online questionnaire including all the instruments, in the same order that we described them in the previous section. In addition, we added a first page with demographic data (e.g., gender and age). Study-related variables such as GPA and time spent studying are included in the open-question sheet of the Studyholism Inventory (SI-10).

Students attending courses in Florence were contacted by means of an invite that they received through their institutional email addresses, thanks to the University Office’s collaboration. In the email that they received, the main objective of the research was explained, and the email address of one of the authors was provided. They could respond through this e-mail address to ask for the link to the questionnaire in order to fill it out anonymously. Since we thought that this kind of active participation (the students contacted us personally in order to get the questionnaire) could limit the participations of students, we tried to reach more Florence students by sharing the link to the questionnaire on several Facebook University groups. Moreover, in order to get the participation of students from other Italian cities and regions, we also shared the questionnaire’s link in Facebook University groups of other Italian cities. The use of social media as a valid recruiting tool is supported by previous studies that have shown that samples collected through this recruitment strategy are no different from those gathered through other data collection methods (105–107).

Given that the questionnaire was administered online, we could not ask the participants to sign the informed consent before filling out the questionnaire. However, we wrote the usual informed consent information in the first page of the questionnaire. Then, we asked the participants to check a box saying that they agreed to take part in the research by going on and filling out the questionnaire on the following pages.

### Data Analysis

We performed the analyses by means of SPPS.22 (Chicago, IL, USA) and AMOS.20 (Chicago, IL, USA).

First, we calculated descriptive statistics for the variables analyzed in this research and their zero-order correlations. Then, in order to test the hypotheses that are related to studyholism and study engagement antecedents and outcomes, we tested a structural equation model (SEM). More specifically, as the model does not involve any latent factor, we tested, by means of path analysis (maximum likelihood estimate method), the direct effects of the antecedents on studyholism and study engagement and on other studyholism antecedents (e.g., worry on study-related perfectionism), as well as the direct effects of studyholism and study engagement on academic, psychological, and physical outcomes and the direct effects of some antecedents on these outcomes. In order to evaluate the fit of the model, we used the following indices and cutoff values: χ^2^/*df* ratio, which indicates a good fit if its value is less than 3 (108), although it is influenced by sample size (109); goodness-of-fit index (GFI), comparative fit index (CFI), Tucker–Lewis index (TLI), and normed fix index (NFI), whose cutoffs are <.90 lack of fit, .90–.95 good fit, and >.95 excellent fit (110); and root mean square error of approximation (RMSEA), where a value below .05 indicates excellent fit, while values between .05 and .08 indicate an acceptable fit (111). For all analyses, *p* < .05 is considered statistically significant and β = ± .10 as the cutoff value for supporting our hypotheses.

Finally, we analyzed if there are antecedents and outcomes differences among the four kinds of student by means of two MANOVAs (which were followed by the Bonferroni *post hoc* test) for a total of 19 follow-up ANOVAs. Due to the high number of multiple comparisons performed to evaluate differences among the four kinds of student on the same sample, we adjusted the alpha level by means of the Bonferroni correction for multiple comparisons. More specifically, we set an adjusted alpha level of .003 (112). The four groups of students have been created referring to the SI-10 (86) cutoff values for Italian College students (85).

## Results

### Structural Equation Model

**Table 3** shows descriptive statistics of all the variables analyzed in the SEM model: studyholism, study engagement, antecedents, and outcomes. **Table 4** shows the zero-order correlations of the study variables. From **Table 3**, it is evident that one variable does not have a normal distribution (quarrels at university); however, we decided to retain it in the model as it is not a predictor and because deleting this scale from the model does not improve the fit markedly.

**Table 3:** Descriptive statistics of all the variables in the model (n = 1,958).

Variable	Range	*M* (SD)	Skewness	Kurtosis
Studyholism	4–20	14.83 (3.78)	−.50	−.47
Study Engagement	4–20	14.77 (3.59)	−.54	−.23
Perfectionistic Concerns (Discrepancy)	3–21	13.27 (4.83)	−.16	.48
Perfectionistic Strivings (Standards)	3–21	16.99 (3.42)	−.85	−.89
Study–related Perfectionism	11–54	28.75 (8.51)	.32	−.42
Worry	16–80	55.21 (14.03)	−.25	−.74
Parent Overstudy Climate	9–45	24.08 (8.98)	.34	−.73
Teacher Overstudy Climate—Hard Study	4–20	8.21 (3.81)	.90	.23
Teacher Overstudy Climate—Overt Comments	5–25	17.43 (4.59)	−.41	−.16
Grade Point Average	18–31*	26.62 (2.22)	−.64	.15
Hours per day of study—generally	0–16	4.47 (2.14)	.60	.50
Hours per day of study—before exams	0–16.5	7.23 (2.35)	.44	.83
Dropout Intention	3–15	6.43 (3.58)	.93	−.19
Positive Affect	10–50	26.99 (8.80)	.21	−.69
Negative Affect	10–50	23.02 (10.22)	.51	−.76
General Stress	0–63	27.78 (16.07)	.28	−.85
Sleep Quality Impairment	5–35	18.63 (7.02)	.09	−.67
Daytime Sleepiness	4–28	18.02 (5.68)	−.30	−.65
Quarrels at University	3–15	4.04 (1.88)	2.46	7.39
Family and Friends’ Complaints	3–15	6.14 (3.05)	.90	.06
Social Relationship Impairment	3–15	7.08 (3.18)	.54	−.51

*Italian GPAs range between 18 and 30; 31 stands for 30 cum laude*.*

**Table 4:** Zero-order correlations for study variables (n = 1,958).

Variable	1	2	3	4	5	6	7	8	9	10	11	12	13	14	15	16	17	18	19	20	21
1. PC	–																				
2. PS	.16***	–																			
3. SPS	.38***	.38***	–																		
4. PSWQ	.48***	.10***	.40***	–																	
5. P-OCS	.20***	−.03	.16***	.16***	–																
6. T-OSC-OC	.22***	.06**	.24***	.19***	.27***	–															
7. T-OSC-HS	.16***	.08***	.20***	.20***	.35***	.52***	–														
8. SH	.42***	.11***	.34***	.73***	.17***	.22***	.25***	–													
9. SE	−.15***	.46***	.29***	.04	−.10***	−.01	−.01	.09***	–												
10. GPA	−.24***	.24***	.21***	−.04	−.15***	−.05*	−.09***	−.08***	.39***	–											
11-H-Gen	.06*	.20***	.12***	.11***	−.03	.06**	.06**	.19***	.30***	.10***	–										
12. H-Exams	.08***	.19***	.18***	.17***	.02	.04	.09***	.20***	.27***	.16***	.56***	–									
13. Drop Int.	.36***	−.13***	.10***	.35***	.19***	.22***	.17***	.36***	−.31***	−21***	-06**	−.04	–								
14. PANAS+	−.20***	.24***	−.02	−.25***	−.09***	−.01	−07**	−.19***	.31***	.09***	.09***	−.01	−23***	–							
15. PANAS−	.44***	.05****	.30***	.57***	.20***	.21***	.17***	.51***	−.08***	−13***	.07**	.10***	.38***	−08***	–						
16. DASS-21	.50***	.07**	.34***	.69***	.23***	.24***	.23***	.62***	−.10***	−13***	.09***	.13***	.47***	−20***	.72***	–					
17. MSQ-SQ	.36***	.07**	.23***	.50***	.19***	.19***	.15***	.46***	-07**	−13***	.05*	.05*	.33***	−10***	.50***	.62***	–				
18. MSQ-DS	.37***	.04	.21***	.46***	.21***	.18***	.19***	.45***	−.09***	−14***	.02	.08***	.31***	−17***	.48***	.60***	.64***	–			
19. SRCS-Q	.18***	.01	.20***	.16***	.18***	.34***	.18***	.15***	−.04	−06**	.04	.05*	.18***	.02	.25***	.25***	.22***	.20***	–		
20. SRCS-C	.08***	.29***	.38***	.26***	.01	.17***	.12***	.26***	.40***	.29***	.30***	.28***	−.02	.15***	.18***	.19***	.12***	.09***	.25***	–	
21. SRCS-SI	.23***	.19***	.35***	.36***	.15***	.20***	.21***	.42***	.22***	.06**	.32***	.32***	.14***	−.03	.30***	.35***	.24***	.23***	.28***	.58***	–

***p ≤.001; **p ≤.01; *p < .05; PC, Perfectionistic Concerns, SAPS Discrepancy scale; PS, Perfectionistic Strivings, SAPS Standard scale; SPS, Study-related Perfectionism Scale; PSWQ, Penn State Worry Questionnaire; P-OCS, Parent Overstudy Climate Scale; T-OSC-OC, Teacher Overstudy Climate Scale, Over Comments scale; T-OSC-HS, Teacher Overstudy Climate, Hard Study scale; SH, Studyholism; SE, Study Engagement; GPA, Grade Point Average; H-Gen, Hours of study per day generally; H-Exams, Hours of study per day before exams; Drop Int., Dropout Intention; PANAS +, Positive Affect; PANAS−, Negative Affect; DASS-21, Depression Anxiety Stress Scale-21, General Stress; MSQ-SQ, Mini Sleep Questionnaire, Sleep Quality; MSQ-DS, Mini Sleep Questionnaire, Daytime Sleepiness; SRCS-Q, Study–Relationships Conflict Scale, Quarrels at School scale; SRCS-C, Study–Relationships Conflict Scale, Family and Friends*’* Complaints scale; SRCS-SI, Study–Relationships Conflict Scale, Social Impairment scale.

**Figure 1:**
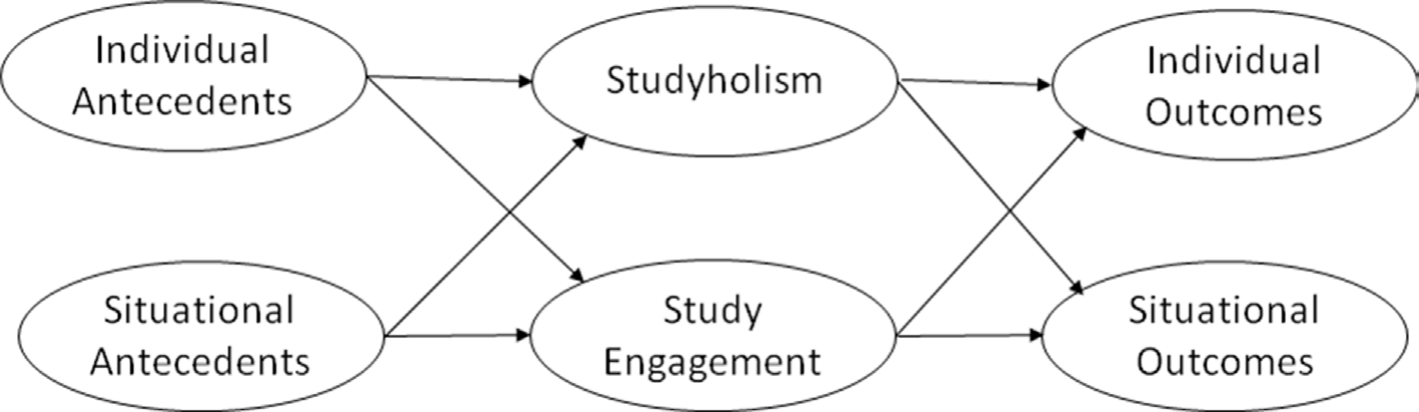
Loscalzo and Giannini’s (2017b) theoretical model.

**Figure 2:**
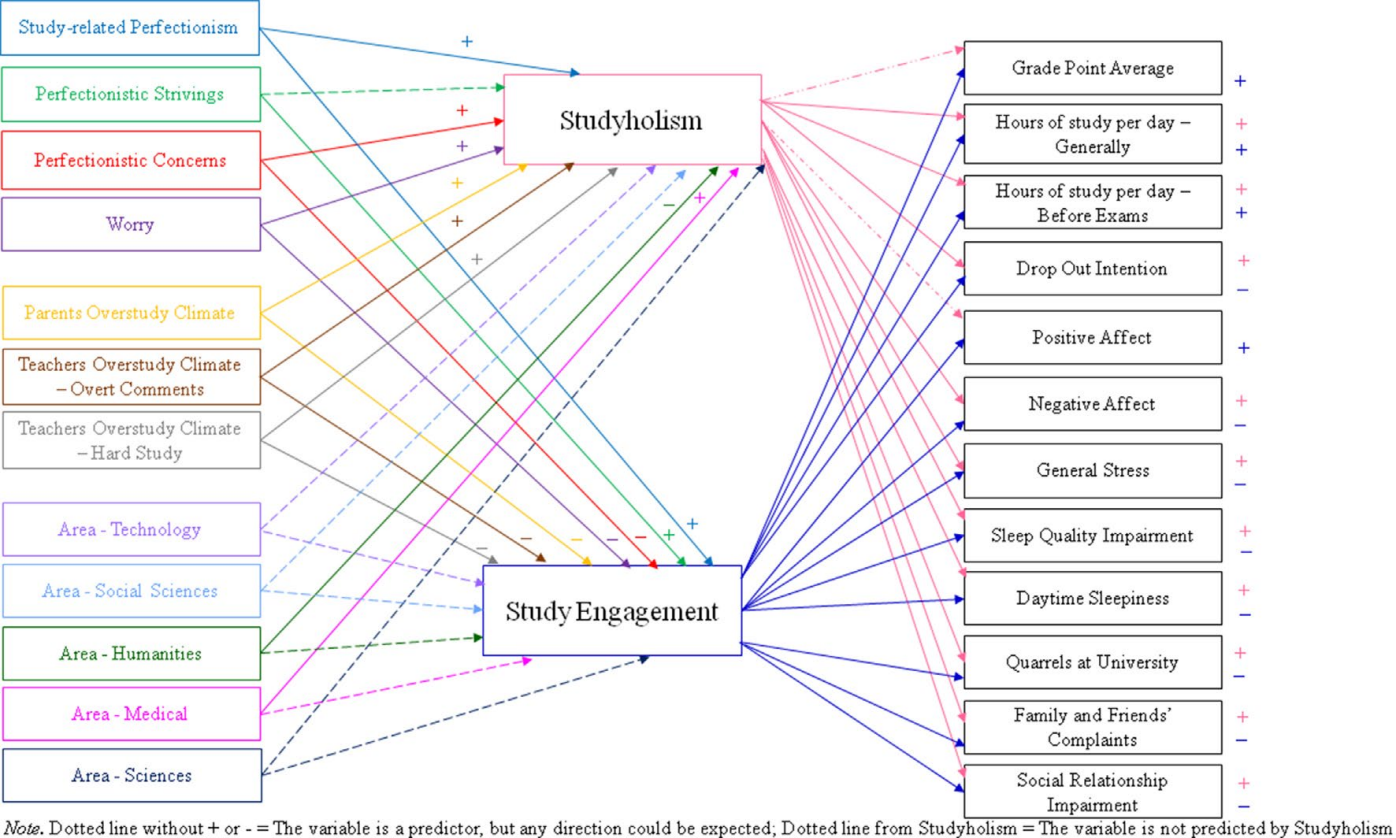
Hypothesized model for Studyholism and Study Engagement antecedents and outcomes.

**Figure 1** depicts the theoretical model we are going to test, namely, Loscalzo and Giannini’s (12) comprehensive model, while **Figure 2** shows the operationalized graphical model as suggested by Nicol and Pexman (113). However, as our hypothesized model involves many antecedents and outcomes, as well as several direct relationships between antecedents and outcomes, we depicted the major links only. Hence, **Figure 2** shows the relationships between both studyholism and study engagement and their antecedents and outcomes.

As far as the other hypothesized relationships are concerned, we presented them in **Table 5**. This table shows all the relationships we are going to analyze with the SEM in order to test our hypotheses about studyholism and study engagement antecedents and outcomes.

**Table 5:** Hypothesized model for studyholism and study engagement antecedents and outcomes (see Hypothesized Direction column) and standardized path weights and *R* ^2^ for each dependent variable of the structural equation model (*n* = 1,958).

Dependent variable	*R* ^2^	Predictor	Hypothesized direction	β	*p*-value	Hypothesis confirmed
Study—Perfectionism	.32					
		Perf. Strivings	+	.32	<.001	Yes
		Perf. Concerns	+	.17	<.001	Yes
		Worry	+	.26	<.001	Yes
		T-OSC-Overt Com.	+	.14	<.001	Yes
Studyholism	.56					
		Perf. Strivings	?	.02	ns	N/A
		Perf. Concerns	+	.06	.001	No
		Study—Perfectionism	+	.02	ns	No
		Worry	+	.67	<.001	Yes
		P-OSC	+	.01	ns	No
		T-OSC-Overt Com.	+	.02	ns	No
		T-OSC-Hard Study	+	.08	<.001	No
		Area—Technology	?	.02	ns	N/A
		Area—Social Sciences	?	−.05	ns	N/A
		Area—Humanities	−	−.08	.01	No
		Area—Medical	+	.01	ns	No
		Area—Sciences	?	−.02	ns	N/A
Study Engagement	.32					
		Perf. Strivings	+	.41	<.001	Yes
		Perf. Concerns	−	−.33	<.001	Yes
		Study—Perfectionism	+	.24	<.001	Yes
		Worry	−	.07	<.001	No
		P-OSC	−	−.07	.002	No
		T-OSC-Overt Com.	−	−.01	ns	No
		T-OSC-Hard Study	−	−.03	ns	No
		Area—Technology	?	−.02	ns	N/A
		Area—Social Sciences	?	−.03	ns	N/A
		Area—Humanities	?	−.02	ns	N/A
		Area—Medical	?	−.02	ns	N/A
		Area—Sciences	?	−.03	ns	N/A
Grade Point Average	.30					
		Perf. Strivings	+	.08	<.001	No
		Perf. Concerns	?	−.25	<.001	N/A
		Study—Perfectionism	+	.21	<.001	Yes
		Studyholism	No	−.08	<.001	Yes*
		Study Engagement	+	.25	<.001	Yes
		Area—Technology	?	.02	ns	N/A
		Area—Social Sciences	?	.09	.02	N/A
		Area—Humanities	?	.34	<.001	N/A
		Area—Medical	?	.13	<.001	N/A
		Area—Sciences	?	.05	ns	N/A
Hours of study per day—Generally	.17					
		Studyholism	+	.15	<.001	Yes
		Study Engagement	+	.30	<.001	Yes
		Study—Perfectionism	+	−.01	ns	No
		Area—Technology	?	.05	ns	N/A
		Area—Social Sciences	?	−.13	.002	N/A
		Area—Humanities	?	−.14	<.001	N/A
		Area—Medical	?	.10	.002	N/A
		Area—Sciences	?	−.02	ns	N/A
Hours of study per day—Before Exams	.15					
		Studyholism	+	.14	<.001	Yes
		Study Engagement	+	.24	<.001	Yes
		Study—Perfectionism	+	.05	.02	No
		Area—Technology	?	.08	.02	N/A
		Area—Social Sciences	?	−.09	.04	N/A
		Area—Humanities	?	−.01	ns	N/A
		Area—Medical	?	.16	<.001	N/A
		Area—Sciences	?	.04	ns	N/A
Dropout Intention	.27					
		Studyholism	+	.30	<.001	Yes
		Study Engagement	−	−.30	<.001	Yes
		Perf. Concerns	+	.18	<.001	Yes
Positive Affect	.19					
		Studyholism	No	−.05	ns	Yes*
		Study Engagement	+	.23	<.001	Yes
		Perf. Strivings	+	.17	<.001	Yes
		Perf. Concerns	−	−.07	.007	No
		Worry	−	−.21	<.001	Yes
Negative Affect	.38					
		Studyholism	+	.20	<.001	Yes
		Study Engagement	−	−.08	<.001	No
		Perf. Concerns	+	.19	<.001	Yes
		Worry	+	.34	<.001	Yes
General Stress	.54					
		Studyholism	+	.25	<.001	Yes
		Study Engagement	−	−.11	<.001	Yes
		Perf. Concerns	+	.18	<.001	Yes
		Worry	+	.43	<.001	Yes
Sleep Quality Impairment	.29					
		Studyholism	+	.21	<.001	Yes
		Study Engagement	−	−.08	<.001	No
		Perf. Concerns	+	.13	<.001	Yes
		Worry	+	.28	<.001	Yes
Daytime Sleepiness	.27					
		Studyholism	+	.24	<.001	Yes
		Study Engagement	−	−.10	<.001	Yes
		Perf. Concerns	+	.15	<.001	Yes
		Worry	+	.22	<.001	Yes
Quarrels at University	.12					
		Studyholism	+	.06	.01	No
		Study Engagement	−	−.08	<.001	No
		Study—Perfectionism	+	.13	<.001	Yes
		T-OSC-Overt Com.	+	.27	<.001	Yes
Family and Friends’ Complaints	.26					
		Studyholism	+	.16	<.001	Yes
		Study Engagement	−	.31	<.001	No
		Study—Perfectionism	+	.24	<.001	Yes
Social Relationship Impairment	.27					
		Studyholism	+	.33	<.001	Yes
		Study Engagement	−	.14	<.001	No
		Study—Perfectionism	+	.20	<.001	Yes
		Area—Technology	?	.08	.004	N/A
		Area—Social Sciences	?	−.04	ns	N/A
		Area—Humanities	?	−.05	ns	N/A
		Area—Medical	?	.08	.004	N/A
		Area—Sciences	?	.06	.03	N/A

+, Positive predictor; −, Negative predictor; No, No predictor;?, No direction specified; β values lower than.10 are judged too low for saying that they support the hypothesis; Perf., Perfectionistic; Study—Perfectionism, Study-related Perfectionism; P-OSC, Parent Overstudy Climate; T-OSC-Overt Com., Teacher Overstudy Climate, Overt Comments; T-OSC-HS, Teacher Overstudy Climate, Hard Study; *, The hypothesis is confirmed, since it stated that the independent variable does not predict the dependent variable; N/A, Not Applicable, there was no specific hypothesis about the positive or negative direction.

**Figure 3:**
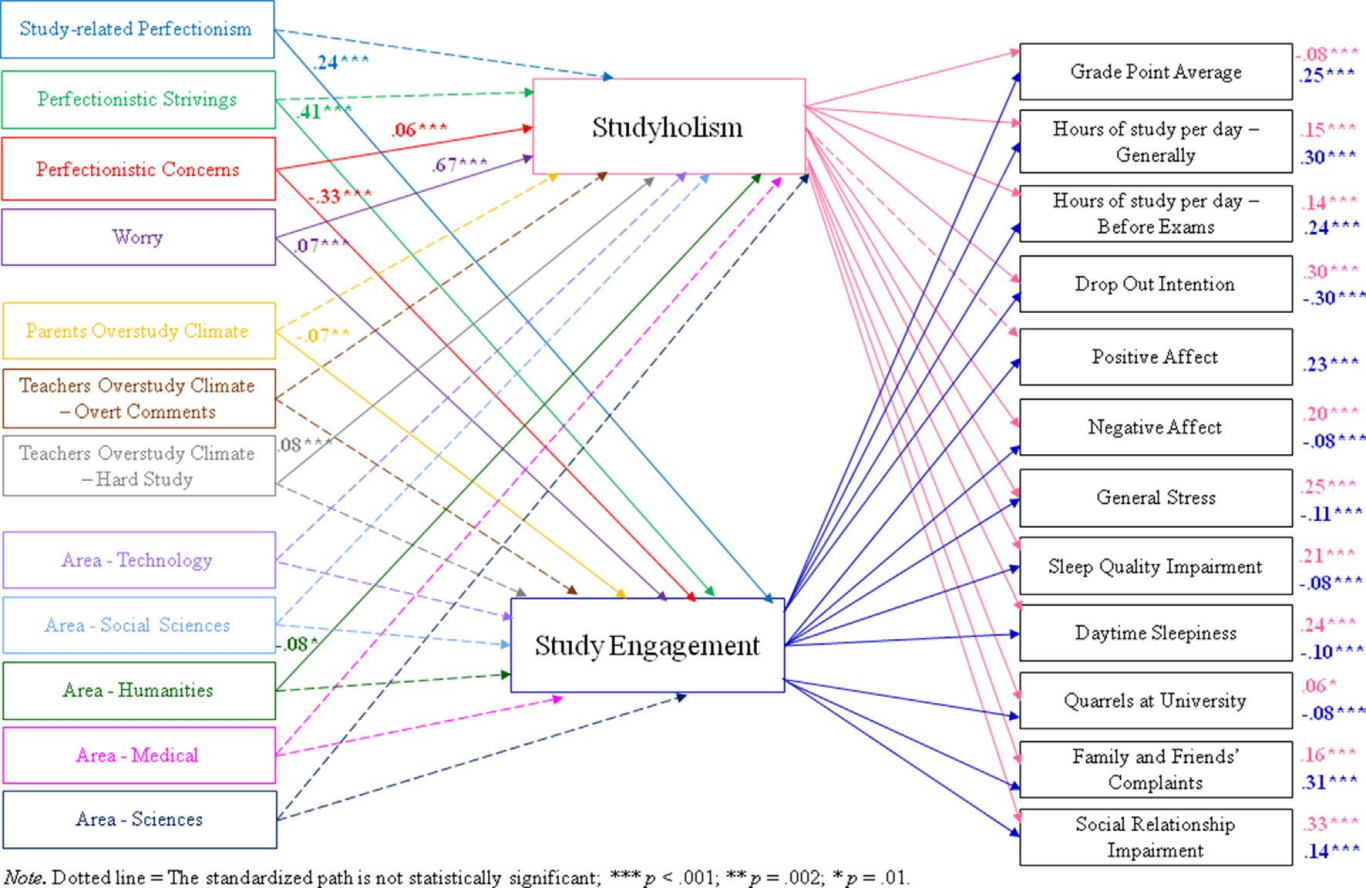
Structural model with standardized path estimates for Studyholism and Study Engagement antecedents and outcomes (n = 1,958).

The hypothesized model showed an excellent fit to the data: χ^2^ = 742.194, *df* = 188, *p* < .001, χ^2^/*df* = 3.95; CFI = .97; NFI = .96; TLI = .95; RMSEA = .039. The structural model, with standardized path estimates and the variance explained by the predictors of each dependent variable, is presented in **Table 5**. Moreover, **Figure 3** depicts the major links with their standardized path estimates.

In sum, the model explains the 56% of the variance in studyholism, with the strongest predictor being worry (β = .67, *p* < .001). The other hypothesized studyholism antecedents are instead not statistically significant or they have very low β values (e.g., PC: β = .06, *p* = .001). Moreover, the model explains 32% of the variance in study engagement, and PS is its strongest predictor (β = .41, *p* < .001).

For outcomes, general stress is the dependent variable whose variance is explained the most by its predictors (they explain the 54% of the variance). The strongest predictor of general stress is worry (β = .43, *p* < .001), followed by studyholism (β = .25, *p* < .001). Finally, the model explains 27% of variance in dropout intention, with studyholism positively predicting it (β = .30, *p* < .001) and study engagement negatively predicting it (β = −.30, *p* < .001).

### Differences in Studyholism Antecedents Among the Four Kinds of Student

In order to analyze if there are differences among the four kinds of student (i.e., disengaged studyholics, engaged studyholics, engaged students, and detached students) in PS, PC, study-related perfectionism, worry, and overstudy climate (P-OSC, T-OSC—overt comments, and T-OSC—hard study), we performed a MANOVA with the type of student as independent variable and the individual and situational antecedents as dependent variables.

**Figure 4:**
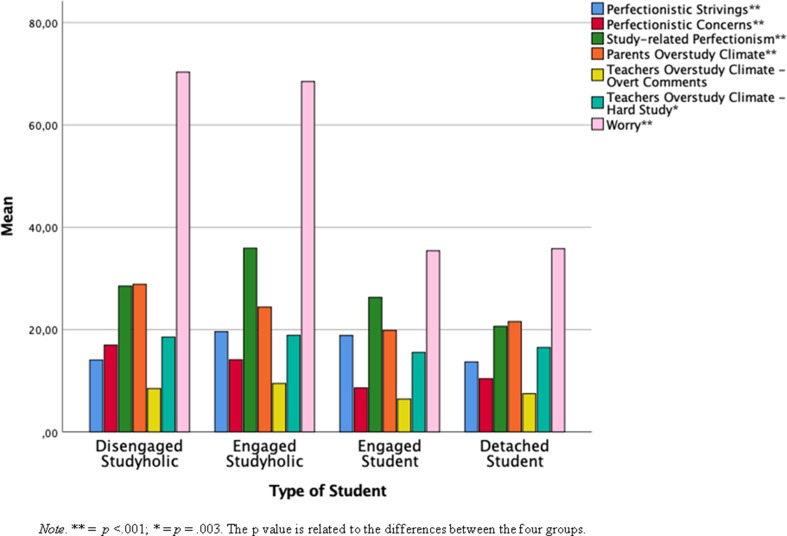
Bar graphs representing the mean of the antecedents by the four groups of student.

The multivariate test showed a statistically significant effect for type of student: *F*(21, 531) = 26.11, *p* < .001, partial η^2^ = .49. Next, follow-up ANOVAs showed that regardless that a Bonferroni adjusted alpha level of .003 is used, there is a statistically significant group difference on all the antecedents analyzed, except for T-OSC—overt comments (*p* = .005). **Table 6** shows the descriptive statistics and the follow-up ANOVA results of MANOVA for all the antecedent variables analyzed. **Figure 4** shows the graphical representation of the contrasts in the means for the four groups analyzed.

**Table 6:** Means (SDs) and follow-up ANOVAs conducted after the MANOVA of antecedents by type of student.

Dependent variable	*F*°	*η* ^2^	*p*	Type of student	*M* (SD)	*n*
Perfectionistic Strivings	42.34	.40	<.001	Disengaged Studyholic	14.06 (4.55)	47
				Engaged Studyholic	19.60 (2.22)	82
				Detached Student	13.69 (4.09)	39
				Engaged Student	18.85 (3.02)	27
				Total	16.98 (4.37)	195
Perfectionistic Concerns	26.02	.29	<.001	Disengaged Studyholic	16.96 (4.22)	47
				Engaged Studyholic	14.11 (4.87)	82
				Detached Student	10.41 (4.11)	39
				Engaged Student	8.63 (4.49)	27
				Total	13.30 (5.33)	195
Study-related Perfectionism	30.68	.33	<.001	Disengaged Studyholic	28.51 (9.82)	47
				Engaged Studyholic	35.93 (8.57)	82
				Detached Student	20.64 (7.05)	39
				Engaged Student	26.30 (8.19)	27
				Total	29.75 (10.35)	195
Worry	173.07	.73	<.001	Disengaged Studyholic	70.32 (8.35)	47
				Engaged Studyholic	68.46 (9.48)	82
				Detached Student	35.82 (12.11)	39
				Engaged Student	35.41 (8.75)	27
				Total	57.81 (18.60)	195
Parent Overstudy Climate	6.75	.10	<.001	Disengaged Studyholic	28.87 (10.10)	47
				Engaged Studyholic	24.40 (10.19)	82
				Detached Student	21.59 (8.43)	39
				Engaged Student	19.85 (6.85)	27
				Total	24.29 (9.85)	195
Teacher Overstudy Climate—	4.38	.06	.005^#^	Disengaged Studyholic	8.49 (4.02)	47
Overt Comments				Engaged Studyholic	9.48 (4.29)	82
				Detached Student	7.49 (4.76)	39
				Engaged Student	6.44 (3.15)	27
				Total	8.42 (4.30)	195
Teacher Overstudy Climate—	4.78	.07	.003	Disengaged Studyholic	18.55 (4.92)	47
Hard Study				Engaged Studyholic	18.89 (4.77)	82
				Detached Student	16.51 (4.95)	39
				Engaged Student	15.56 (4.01)	27
				Total	17.87 (4.89)	195

°, df = 3,191; ^#^, using Bonferroni correction, it is not statistically significant.

More specifically, the Bonferroni *post hoc* test revealed that disengaged studyholics have statistically significantly lower levels of *PS* than have both engaged studyholics (*p* < .001) and engaged students (*p* < .001). Moreover, engaged studyholics and engaged students have higher PS than have detached students (*p* < .001). There is no statistically significant difference between disengaged studyholics and detached students and between engaged studyholics and engaged students.

With regard to *PC*, disengaged studyholics have statistically significantly higher levels than have engaged studyholics (*p* = .004), detached students (*p* < .001), and engaged students (*p* < .001). Moreover, engaged studyholics have statistically significantly (*p* < .001) higher PC than have detached and engaged students. There is no difference between detached students and engaged students.

In addition, engaged studyholics have statistically significantly (*p* < .001) higher levels of *study-related perfectionism* than have disengaged studyholics, and engaged and detached students. Moreover, disengaged studyholics have statistically significantly (*p* < .001) higher levels of study-related perfectionism than have detached students. Finally, detached students have marginally significantly (*p* = .054) lower levels of this antecedent than have engaged students. There is no difference between disengaged studyholics and engaged students.

Next, disengaged studyholics and engaged studyholics scored higher on *worry* than did both detached and engaged students (*p* < .001). There is no difference between disengaged and engaged studyholics, and between detached and engaged students.

Finally, concerning the overstudy climate, Bonferroni *post hoc* analyses showed, for *P-OSC*, that disengaged studyholics have statistically significantly higher levels than have detached (*p* = .003) and engaged (*p* = .001) students; however, there is no difference between disengaged and engaged studyholics, and there are no other statistically significant group difference on this variable. Moreover, the only statistically significant (*p* = .011) group difference on *T-OSC—hard study* is between engaged studyholics and engaged students, with the former scoring higher.

### Differences in Studyholism Outcomes Among the Four Kinds of Student

In order to investigate if there are differences among the four kinds of student in sleep quality impairment, daytime sleepiness, positive and negative affect, general stress, quarrels at university, family and friends’ complaints, social relationship impairment due to study, time investment in study, GPA, and intention to drop out from university, we performed a MANOVA with type of student as the independent variable and the outcomes as dependent variables.

**Figure 5:**
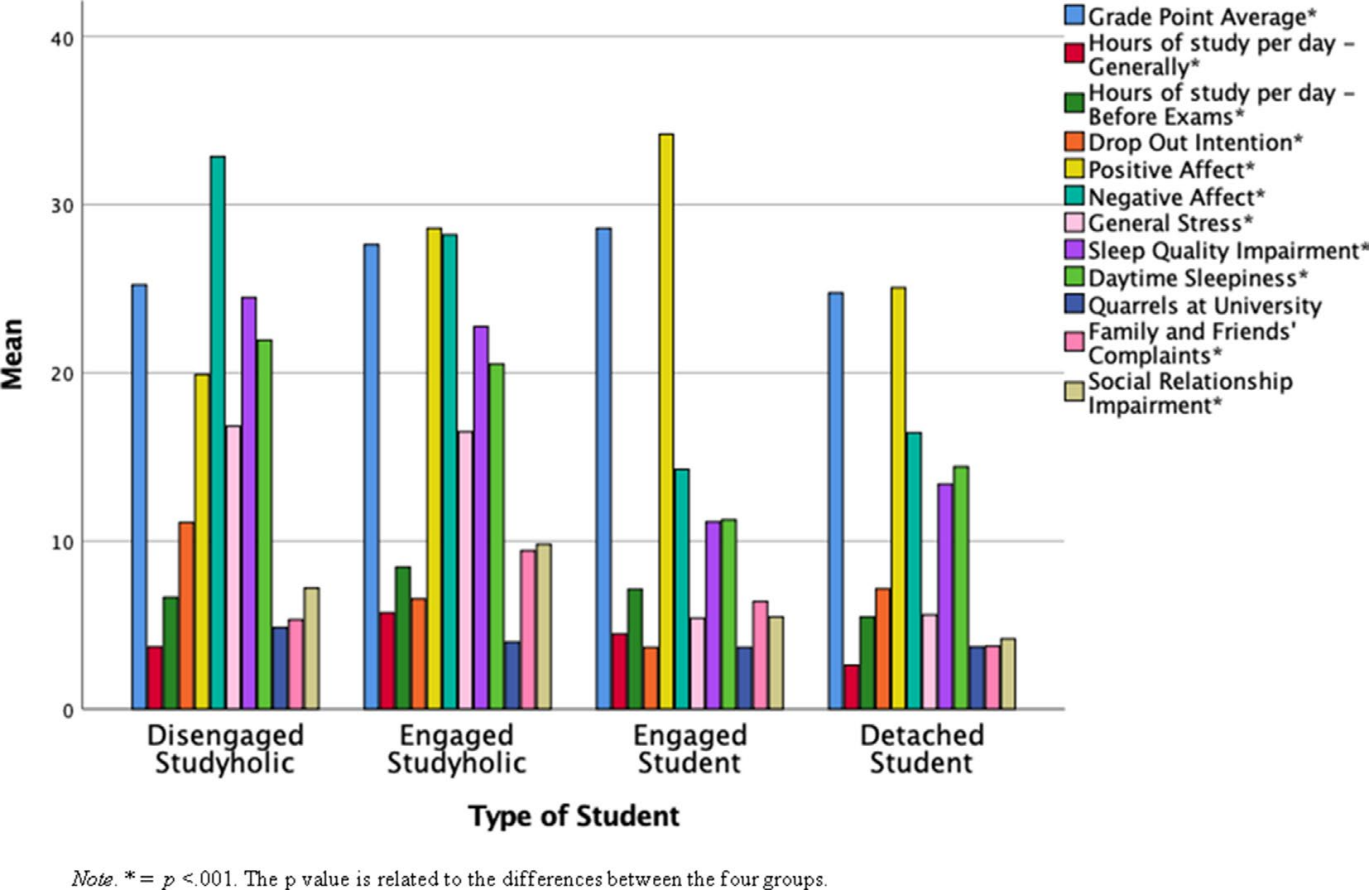
Bar graphs representing the mean of the outcomes by the four groups of students.

The multivariate test showed a statistically significant effect for type of student: *F*(36, 532) = 16.97, *p* < .001, partial η^2^ = .53. Next, follow-up ANOVAs showed that, regardless that a Bonferroni adjusted alpha level of .003 is used, there is a statistically significant group difference on all the outcomes analyzed, except for quarrels at university (*p* = .035). **Table 7** shows the descriptive statistics and the subsequent ANOVAs conducted after the multivariate test. **Figure 5** shows the graphical representation of the contrasts in the means for the four groups analyzed.

**Table 7:** Means (SDs) and follow-up ANOVAs conducted after the MANOVA of outcomes by type of student.

Variable	*F*°	*η* ^2^	*p*	Type of student	*M* (SD)	*n*
Sleep Quality^§^	45.88	.42	<.001	Disengaged Studyholic	24.47 (6.80)	47
				Engaged Studyholic	22.74 (6.77)	82
				Detached Student	13.38 (4.79)	39
				Engaged Student	11.15 (5.26)	27
				Total	19.68 (8.12)	195
Daytime Sleepiness	29.43	.32	<.001	Disengaged Studyholic	21.94 (5.25)	47
				Engaged Studyholic	20.52 (6.11)	82
				Detached Student	14.41 (5.55)	39
				Engaged Student	11.26 (5.98)	27
				Total	18.36 (6.94)	195
Positive Affect	17.85	.22	<.001	Disengaged Studyholic	19.89 (7.94)	47
				Engaged Studyholic	28.59 (9.33)	82
				Detached Student	25.05 (7.81)	39
				Engaged Student	34.19 (9.41)	27
				Total	26.56 (9.81)	195
Negative Affect	39.92	.39	<.001	Disengaged Studyholic	32.85 (10.72)	47
				Engaged Studyholic	28.21 (9.75)	82
				Detached Student	16.44 (6.99)	39
				Engaged Student	14.26 (4.98)	27
				Total	25.04 (11.40)	195
General Stress	100.88	.61	<.001	Disengaged Studyholic	16.83 (3.90)	47
				Engaged Studyholic	16.49 (4.66)	82
				Detached Student	5.62 (3.57)	39
				Engaged Student	5.41 (4.13)	27
				Total	12.86 (6.72)	195
Quarrels at University	2.92	.04	.035^#^	Disengaged Studyholic	4.85 (3.22)	47
				Engaged Studyholic	3.99 (1.60)	82
				Detached Student	3.69 (1.58)	39
				Engaged Student	3.67 (1.71)	27
				Total	4.09 (2.14)	195
Family Friends’ Compl.	42.34	.40	<.001	Disengaged Studyholic	5.32 (3.51)	47
				Engaged Studyholic	9.41 (3.08)	82
				Detached Student	3.74 (1.37)	39
				Engaged Student	6.41 (2.34)	27
				Total	6.88 (3.65)	195
Social Rel. Impairment	33.20	.34	<.001	Disengaged Studyholic	7.19 (3.72)	47
				Engaged Studyholic	9.80 (3.28)	82
				Detached Student	4.18 (2.15)	39
				Engaged Student	5.48 (2.76)	27
				Total	7.45 (3.84)	195
Hours—Generally	20.52	.24	<.001	Disengaged Studyholic	3.69 (2.34)	47
				Engaged Studyholic	5.73 (2.34)	82
				Detached Student	2.61 (1.52)	39
				Engaged Student	4.48 (2.18)	27
				Total	4.44 (2.49)	195
Hours—Before Exams	12.66	.17	<.001	Disengaged Studyholic	6.65 (2.62)	47
				Engaged Studyholic	8.45 (2.48)	82
				Detached Student	5.49 (2.82)	39
				Engaged Student	7.13 (2.60)	27
				Total	7.24 (2.83)	195
Grade Point Average	32.49	.34	<.001	Disengaged Studyholic	25.24 (2.41)	47
				Engaged Studyholic	27.62 (1.89)	82
				Detached Student	24.75 (2.32)	39
				Engaged Student	28.59 (1.40)	27
				Total	26.61 (2.51)	195
Dropout Intention	31.95	.33	<.001	Disengaged Studyholic	11.11 (3.36)	47
				Engaged Studyholic	6.56 (3.80)	82
				Detached Student	7.15 (3.38)	39
				Engaged Student	3.67 (1.14)	27
				Total	7.37 (4.09)	195

°, df = 3,191; ^#^, using Bonferroni correction, it is not statistically significant. ^§^, A higher score indicates greater sleep quality impairment; Family Friends Compl., Family and Friends’ Complaints; Social Rel. Impairment, Social Relationship Impairment; Hours—Generally, Hours of study per day, Generally; Hours—Before Exams, Hours of study per day, Before exams.

More specifically, concerning *sleep quality impairment* and *daytime sleepiness*, the Bonferroni *post hoc* test revealed that disengaged and engaged studyholics have statistically significantly (*p* < .001) higher levels of sleep quality impairment and daytime sleepiness than have detached and engaged students; however, there is no difference between engaged and disengaged studyholics and between engaged and detached students. Hence, disengaged studyholics do not significantly differ from engaged studyholics; however, they both have greater sleep impairment than have engaged and detached students.

Next, with regard to *positive and negative affect*, disengaged studyholics have statistically significantly lower levels of positive affect than have engaged studyholics (*p* < .001), engaged students (*p* < .001), and detached students (*p* = .04). Moreover, engaged studyholics (*p* = .026) and detached students (*p* < .001) have lower positive affect than have engaged students. There is no difference between engaged studyholics and detached students. For negative affect, disengaged and engaged studyholics have statistically significantly (*p* < .001) higher level than have detached and engaged students. In addition, disengaged studyholics have higher negative affect than have engaged studyholics (*p* = .032). There is no difference between engaged and detached students. In sum, disengaged studyholics have the lowest levels of positive affect and the highest levels of negative affect.

About *general stress*, the results showed that disengaged and engaged studyholics have statistically significantly (*p* < .001) higher general stress than have engaged and detached students. There is no difference between disengaged and engaged studyholics, nor between engaged and detached students. Thus, there is no difference on general stress between disengaged and engaged studyholics.

Then, the Bonferroni *post hoc* test showed that engaged studyholics have significantly (*p* < .001) higher levels of *family and friends*’* complaints* than have disengaged studyholics, and engaged and detached students. Also, detached students have lower levels of family and friends’ complaints than have engaged students (*p* < .001). There is no difference between disengaged studyholics and both detached and engaged students. For *social relationship impairment*, engaged studyholics have higher relationship impairment (*p* < .001) than have disengaged studyholics and detached and engaged students. Moreover, disengaged studyholics have higher social relationships impairment than have detached students (*p* < .001). There is no difference between disengaged studyholics and engaged students, and between detached and engaged students. In sum, there is no statistically significant difference between the four kinds of student concerning quarrels at university. However, engaged studyholics have higher levels of family and friends’ complaints and of social relationship impairment than have disengaged studyholics.

Next, concerning academic-related outcomes, we found that engaged studyholics generally study more *hours per day* than do disengaged studyholics and detached students (*p* < .001), and that engaged students study more hours a day than do detached students (*p* = .004). There is no difference between disengaged studyholics and both detached students and engaged students, nor between engaged studyholics and engaged students. Moreover, as far as *hours of study per day before exams* are concerned, engaged studyholics study more hours than do disengaged studyholics (*p* = .001) and detached students (*p* < .001). There are no differences between disengaged studyholics and both detached and engaged students, nor between engaged studyholics and engaged students, nor finally between detached students and engaged students. Hence, engaged studyholics spend more time studying than do disengaged studyholics.

Moreover, for *GPA*, disengaged studyholics have statistically significantly (*p* < .001) lower GPA than have engaged studyholics and engaged students. Moreover, engaged studyholics have higher GPAs than have detached students (*p* < .001). Finally, engaged students have higher GPAs than have detached students (*p* < .001). There is no difference between disengaged studyholics and detached students, and between engaged studyholics and engaged students. Hence, disengaged studyholics have statistically significantly lower GPAs than have engaged studyholics.

Finally, for *dropout intention*, disengaged studyholics have statistically significantly (*p* < .001) higher dropout intention than have engaged studyholics, engaged students, and detached students. Moreover, engaged studyholics (*p* = .001) and detached students (*p* < .001) have higher dropout intention than have engaged students. In addition, detached students have higher dropout intention than have engaged students (*p* < .001). There is no difference between engaged studyholics and detached students. In sum, disengaged studyholics have higher dropout intention than have engaged studyholics.

## Discussion

### Individual Antecedents

The first hypothesis concerns *PS*, and the SEM results supported almost all of the relationships suggested about this antecedent.

As hypothesized, PS, as general perfectionism, positively predicts study-related perfectionism, which is rather a domain-specific type of perfectionism. Moreover, in line with the literature (45–47), it also positively predicts study engagement (SE). However, it does not predict studyholism (SH), in line with Loscalzo (84) preliminary study, but in contrast with the workaholism literature (45). This finding may suggest that this positive component of perfectionism should not be addressed by interventions aimed to prevent or to reduce studyholism, as it does not seem to foster it. Moreover, as workaholism is instead usually associated with high PS, we speculate that workaholism and studyholism, even though they both are related to the main work activity of workers and students, respectively, could differ in some aspects, for example, in their antecedents. However, it should be noted that even though workaholism studies have generally found a positive association between PS and workaholism, Mazzetti et al. (49) recently highlighted that this relationship emerges only when workers perceive an overwork climate in their organization. Hence, as our study is the first one dealing with studyholism and PS, we suggest that future studies should be conducted on this topic and that no solid conclusion can be made yet.

Finally, PS positively predicts positive emotions, in line with previous studies related to positive health outcomes associated with PS [e.g., Refs. (32, 36, 38)]. However, we did not find support for a relationship between PS and GPA. While previous studies found a positive association between these two variables (52), we found that PS is a statistically significant positive predictor of GPA, but with very low magnitude, which is near zero. Since in the model we tested many other variables besides PS, a possible explanation for this low (although positive) value is that the other variables included in the model explain GPA better than PS; for example, SE positively predicts it with a β value of .25.

For *PC*, this general form of perfectionism positively predicts study-related perfectionism, as hypothesized. However, it predicts this domain-specific type of perfectionism with a lower value than does PS. Moreover, as with PS, it does not predict SH. The β value is positive and statistically significant, but since it is near to zero, it does not allow us to conclude that the hypothesis is supported. Hence, this result contrasts with the workaholism literature, which has generally found a positive association between PC and workaholism (45), and with Loscalzo and Giannini (12), who suggested in their theoretical paper that perfectionism is a studyholism antecedent. Again in this case, we suggest that future studies should analyze the relationship between PC and studyholism, as this could inform whether our result is due to the instrument we used for evaluating perfectionism, or to an actual difference between studyholism and workaholism concerning their antecedents. Indeed, it should be noted that the Italian version of the instrument that we used to measure perfectionism, namely, the SAPS (90), does not have a fully satisfactory fit in the Italian version. Hence, even if this study has the merit of shedding some light on the relationships among PS, PC, studyholism, and study engagement, we suggest that future studies should focus more on these relationships and use other perfectionism instruments, in order to deepen the analysis of this topic.

In addition, we did not find support for the hypothesis regarding positive affect. PC negatively predicts positive affect, although its β value is too low for concluding that the hypothesis has been supported. However, the other hypotheses have been confirmed. PC negatively predicts SE, as hypothesized based on the negative association generally found between work engagement and PC (45). It also positively predicts dropout intention, even though studyholism and study engagement have, respectively, a positive and negative higher predictive power than PC on dropout intentions.

In addition, PC positively predicts negative affect, general stress, sleep quality impairment, and daytime sleepiness, as hypothesized based on the literature about PC and wellbeing [e.g., Refs. 31, 33)] (114).

Finally, regarding GPA, we did not posit any specific hypothesis, as the literature showed mixed findings about the relationship between PC and GPA. This study found that PC significantly and negatively predicts GPA, and it is one of the strongest predictors of GPA.

In sum, it seems that this perfectionism component also should not be addressed by preventive and clinical interventions targeting studyholism, as it does not appear to foster it. However, as already stated for PS, further studies should be conducted on these relationships before any conclusion should be made. Moreover, PC is an individual antecedent that deserves to be addressed by preventive interventions aiming to enhance students’ wellbeing and academic success, as it is the strongest predictor of low academic performance (GPA); moreover, even though it does not negatively predict positive emotions, it negatively predicts study engagement (which is usually associated with positive academic performance and wellbeing), and it positively predicts dropout intention and all of the physical and psychological negative outcomes that are included in the model. Hence, even if it does not seem to foster studyholism, it is an individual component that should potentially be targeted in order to reduce dropout intention and increase study engagement.

Next, in the perfectionism domain, the results about *study-related perfectionism* showed that, contrary to the hypothesis, it does not predict studyholism and time spent studying (hours of study per day generally and before exams), but it does positively predict study engagement, GPA, quarrels at university, family and friends’ complaints, and social relationship impairment due to study. Hence, study-related perfectionism seems to be an antecedent of positive attitudes toward studying (i.e., study engagement) and good academic performance. However, we suggest not attempting to foster it by means of interventions that aim to favor academic success, as one of its predictor is PC, which is usually associated with negative outcomes; moreover, study-related perfectionism is also associated with more quarrels with teachers and peers at university, with more complaints from family and friends and relationship impairment due to study. Hence, besides its positive association with study engagement and GPA, it is associated with negative outcomes as well, especially as far as social functioning is concerned. For this reason, we suggest that preventive interventions aimed at enhancing academic success and students’ wellbeing should address study-related perfectionism with the aim of reducing it, and also foster instead other variables that are associated with positive outcomes only, such as study engagement. Since study-related perfectionism is associated with higher GPA, students might see it as a positive characteristic that they should not dismiss; for this reason, they should be made aware of the negative downsides associated with such perfectionism and that they may be able to gain the same academic success by increasing other personal characteristics that are associated with positive outcomes, such as study engagement.

In addition to perfectionism, there is another individual antecedent that we analyzed by means of the SEM, namely, *trait worry*. First, as hypothesized on the basis of the literature showing that worry is related to perfectionism [e.g., Refs. (79, 80)], we found that it positively predicts study-related perfectionism. Contrary to the hypothesis, it is not a negative predictor of study engagement, but *it is the strongest predictor of studyholism* (β = .67, *p* < .001), consistent with the literature showing that worry is a factor contributing to OCD (68) and a transdiagnostic process across internalizing disorders [e.g., Refs. (64, 66)]. Finally, in line with previous studies showing an association between worry and health impairment [e.g., Refs. (75, 76)], it negatively predicts positive affect, while it positively predicts negative affect, general stress, sleep quality impairment, and daytime sleepiness. Hence, these results confirm that trait worry predicts many negative physical and psychological outcomes. Moreover, it demonstrated that it is a strong predictor of studyholism, suggesting that it should be the primary focus of interventions aiming to prevent and to reduce studyholism. In addition, from a theoretical point of view, this suggests that studyholism is an internalizing disorder, or an OCD-related disorder, as suggested by Loscalzo and Giannini (12, 14, 15), and not a behavioral addiction (11). Future studies should deepen our understanding of the role of trait worry, and of other internalizing features, in the onset of studyholism.

### Situational Antecedents

Besides individual antecedents, we also analyzed two situational antecedents.

For *school and family overstudy climate*, contrary to our hypothesis, none of the three overstudy climate factors (i.e., P-OSC, T-OSC—overt comments, and T-OSC—hard study) predicts studyholism and study engagement, even though the direction is positive for SH and negative for SE, as expected. Hence, we conclude that overstudy climate does not foster studyholism and reduce study engagement in university students. However, we suggest that this situational antecedent should be analyzed in secondary school of first and second grades (i.e., pre-adolescence and adolescence), namely, when studyholism could have its onset (12), when students are younger and hence could be more dependent on what elder significant people expect from them. Finally, as far as overstudy climate is concerned, the results showed that, as hypothesized, teachers’ overt comments about students’ performance positively predict two study-related variables, namely, study-related perfectionism and aggressive behaviors at the university in the specific form of quarrels. Thus, this suggests that teachers, even if they do not foster studyholism or impair study engagement by means of their overt comments about students’ performance (e.g., asking for explanations when they get a grade lower than usual), they can increase quarrels between students and between student and teacher, hence affecting the class climate. For this reason, preventive interventions aimed at supporting positive relationships at the university should address this overstudy climate component by means of educational interventions for teachers, who should be made aware of the potential counterproductive effects of their overt comments in class. These negative effects could be even greater in the lower school levels, where there are fewer students in class and group dynamics are more evident (12).

Another situational antecedents is *area of study*, which in this research has been grouped into the following macro-groups comprising many different courses: technology (engineering, architecture, and informatics); social sciences (psychology, sociology, economy, law, educational studies, etc.); humanities (literature, language, art, philosophy, history, etc.); medical studies; sciences (math, physics, biology, statistics, and chemistry); helping professions (nursing, obstetrics, etc.); and para-medical studies (biotechnology, veterinary medicine, pharmacy, etc.).

Our hypotheses were not confirmed. More specifically, humanities students have statistically significantly lower levels of studyholism than have the other groups; however, the β value is near to zero; thus, while in the expected direction, this does not allow concluding that the hypothesis has been confirmed or supported. As far as the other areas of study are concerned, the results showed that technology, social sciences, and sciences do not predict studyholism, as well as medical and humanities studies. Moreover, the five areas of study do not predict study engagement as well.

Moreover, the results showed that both humanities and medical students have a higher GPA than have their peers. However, humanities and social sciences students generally spend less hours per day in studying than do their peers in other courses, while medical students study more hours per day both generally and, even more, before exams. No other statistically significant relationship emerged (considering .10 as the β cutoff value). For social relationship impairment due to study, medical, technology, and sciences students have statistically significantly higher impairment than have their peers, but the β values are below .10. In conclusion, it seems that medical students spend more time studying than their peers do, and they receive higher GPAs. However, the students with higher GPAs are humanities students, who also spend less time studying (as well as social sciences students).

On the basis of these findings, we suggest that studyholism preventive interventions should not address a specific area of study, as there is no specific area that positively predicts higher levels of studyholism, but they should rather be distributed across all the courses.

### Academic, Health, and Social Outcomes

As concerns the *academic outcomes*, the results showed that, as hypothesized, studyholism does not predict GPA. In line with previous studies on study addiction and studyholism (11, 85), studyholism has only a low negative predictive value on GPA. Study engagement, instead, positively predicts this variable, in line with previous studies [e.g., Refs. (16, 17, 85)]. Also, in line with Loscalzo and Giannini (12, 85), both studyholism and study engagement positively predict time investment in study, both generally and before exams, respectively. Finally, as hypothesized and in line with Loscalzo and Giannini’s (12) model and with van Beek et al.’s (25) workaholism study, SH positively predicts dropout intention, while SE negatively predicts it, in line with the previous literature [e.g., Refs. (16, 21)].

The practical implication of these findings is that s*tudyholism is an important risk factor for the drop out of University, while study engagement is a protective factor*. Moreover, as SH and SE are both associated with higher time investment in study but only SE is positively related to higher GPA too, it follows that preventive interventions should focus on training students to learn how to organize their time investment in study fruitfully, and how to reduce their study-related obsessions that could actually lead them to overstudy but without actual learning, or to study a few pages well in many hours. In addition, preventive and clinical interventions should increase study engagement, as it increases the probability of finishing one’s own studies.

Next, as far as *psychological individual outcomes* are concerned, the SEM analyses showed that studyholism does not predict positive affect, in line with the hypothesis. However, as hypothesized, it positively predicts negative emotion and general stress, in line with the previous studyholism and workaholism literature [e.g., Refs. (5, 11, 12)]. Study engagement, instead, positively predicts positive affect and negatively predicts general stress, in line with previous studies [e.g., Ref. (19)]. However, it negatively predicts negative affect with a low β value; since it does not reach the selected .10 cutoff, we suggest that this hypothesis has not been confirmed or that study engagement is not an important variable for the explanation of lower levels of negative emotions.

Then, concerning *individual physical outcomes*, studyholism positively predicts sleep quality impairment and daytime sleepiness, in line with the hypothesis and previous studies (5, 11). Study engagement, in contrast, negatively predicts both the two variables, but the β value reaches the .10 cutoff for daytime sleepiness only. Hence, these results suggest that studyholism is associated with negative health outcomes; therefore, preventive and clinical interventions specifically developed for reducing studyholism should be conducted, as it is associated with significant health impairment.

Regarding *social relationships*, and more specifically aggressive behaviors at university, which represent a situational outcome, the results showed that neither studyholism nor study engagement predicts it, in contrast to the hypothesis. The direction is, as expected, positive for SH and negative for SE, although the β values do not reach the .10 cutoff. As already mentioned for overstudy climate, we suggest, in line with Loscalzo and Giannini (12), that aggressive behaviors in class could be more prevalent in non-university schools, as there are closer relationships between peers and between students and teachers. Hence, future studies should analyze the impact of studyholism on aggressive behaviors in pre-adolescents and adolescents.

Next, concerning individual outcomes related to social relationships, the results showed that studyholism positively predicts both family and friends’ complaints and social relationship impairment because of study, in line with previous studies on workaholism (5), and with Loscalzo and Giannini’s (12) model. However, in contrast with the hypothesis, study engagement, too, positively predicts both family and friends’ complaints and social relationship impairment because of study. Hence, these results could suggest that heavy study investment (regardless of whether it is studyholism or study engagement) is not a generally social acceptable behavior, as previously hypothesized similarly to workaholism (12), especially considering that the β value for family and friends’ complaints is higher for study engagement. Moreover, heavy study investment leads to relationship impairment also in the case of study engagement, even if to a lesser extent than does studyholism. This means that preventive interventions aiming to foster students’ wellbeing, and not only their academic success, might well be addressed to engaged students as well as studyholics, as they are both characterized by social impairment. They should receive some training with the aim of improving their management of time and hence being able to keep on studying and also being able to free some of their time for friends, family, and leisure activities.

### Differences Between Disengaged and Engaged Studyholics

For perfectionism, the results showed that *PS* is statistically significantly lower in disengaged studyholics as compared with both engaged studyholics and engaged students. Moreover, engaged studyholics and engaged students have statistically significantly higher PS than have detached students. In sum, the positive component of perfectionism is present at the lowest levels in detached students, while the highest (mean) level is in engaged studyholics. This could be due to the fact that standards are very high both in studyholics and in engaged students, and that as a consequence PS reaches its highest levels in students characterized by both high SH and SE. Looking at the descriptive statistics, the following is the order as concerns PS levels: detached students < disengaged studyholics < engaged students < engaged studyholics.

Then, regarding the negative perfectionism component, disengaged studyholics have statistically significantly higher levels of *PC* than have all the other kinds of student. In addition, engaged studyholics have higher levels of PC than have detached and engaged students. Referring to the descriptive statistics, the following is the order of PC levels: engaged students < detached students < engaged studyholics < disengaged studyholics. Hence, the negative perfectionism component is present at its highest levels in disengaged studyholics, while at the lowest level, it is in engaged students. However, both disengaged and engaged studyholics have higher PC than have the other two kinds of student.

Finally, for *study-related perfectionism*, engaged studyholics have statistically significantly higher levels of this than have the other three kinds of student. Moreover, disengaged studyholics have statistically significantly higher study-related perfectionism than have detached students. Referring to the descriptive statistics, the order is the following: detached students < engaged students < disengaged studyholics < engaged studyholics.

Taken together, these results support Loscalzo and Giannini’s (12) proposition about the need for distinguishing among the three kinds of heavy study investor, and that the engaged student is the most positive type, since it has the lowest levels of PC. The disengaged studyholics, instead, are the most negative type, being characterized by the highest levels of PC.

Next, regarding *overstudy climate*, disengaged studyholics have higher P-OSC than have detached and engaged students, but there is no statistically significant difference from engaged studyholics. For T-OSC, there is no statistically significant difference for overt comments about students’ performance; however, engaged studyholics scored higher on pressure toward hard study than had engaged students, but there is no statistically significant difference between engaged and disengaged studyholics. Looking at the descriptive statistics, the order for P-OSC is engaged students < detached students < engaged studyholics < disengaged studyholics, while for T-OSC (for both its two components), the order is engaged students < detached students < disengaged studyholics < engaged studyholics. However, as already mentioned before, the analysis related to overstudy climate should be further conducted on pre-adolescents and adolescents, where the influence of this climate could be greater than on youths.

Finally, concerning *worry*, disengaged studyholics and engaged studyholics have statistically significantly higher worry levels than have detached and engaged students, but there is no difference between disengaged and engaged studyholics. However, looking at their means, disengaged studyholics score a little higher than do engaged studyholics. Hence, these results suggest that engaged and disengaged studyholics do not differ in worry, but they do score higher than the other two types of students.

About studyholism *outcomes*, the results showed that engaged and disengaged studyholics have more sleep quality impairment, daytime sleepiness, and general stress than have detached and engaged students, but (besides a slight difference in their means) there is no statistically significant difference between disengaged and engaged studyholics.

However, disengaged studyholics have lower positive affect than have all the other three kinds of student, and engaged studyholics have lower positive emotions than have engaged and detached students. Moreover, engaged and disengaged studyholics have higher negative affect than have detached and engaged students; however, also in this case, disengaged studyholics are more impaired, as they have higher negative affect than have engaged studyholics. Hence, as far as positive and negative affect is concerned, disengaged studyholics have the highest levels of negative affect and the lowest levels of positive affect.

However, the engaged studyholics have higher family and friends’ complaints and social relationship impairment than have all the other three types of student. Moreover, there are no group differences related to quarrels at university.

Hence, based on these results, it seems that disengaged studyholic are not more impaired than engaged studyholics [as suggested by Loscalzo and Giannini (12)]. The two types of studyholics do not differ in sleep quality and stress experienced. Moreover, while disengaged studyholics are more emotionally impaired than are engaged studyholics, engaged studyholics are the ones more socially impaired.

Finally, for *study-related variables*, disengaged studyholics have higher dropout intention than have the other three types of student, while engaged studyholics have higher dropout intention than have engaged students. Moreover, disengaged studyholics have a lower GPA than have engaged studyholics and engaged students, while engaged studyholics have a higher GPA than have detached students. Hence, as concerns academic outcomes, both disengaged and engaged studyholics show a functional impairment; however, disengaged studyholics are the most impaired [in line with Loscalzo and Giannini (12)].

Concerning time spent studying, engaged studyholics study more hours a day (generally and before exams) than do disengaged studyholics and detached students. Moreover, engaged students generally study more than detached students do (but not before exams). Hence, in line with the workaholism study of Van Beek et al. (25), engaged studyholics are the ones who spend the most time studying. This could be due to the presence of both high studyholism and study engagement, and this could also explain why, contrary to the hypothesis, these are also the most socially impaired students in this study. It is possible to suggest that they are not able to manage their time; hence, the time they spend studying is useful for having a higher GPA as compared with that of disengaged studyholics, but they are not able to leave some time free for meeting friends and spending time with their families.

In conclusion, it is useful to differentiate between disengaged and engaged studyholism, and more generally between the four kinds of student, as this allows us to unpack different relationships with the same antecedents and outcomes. In sum, PC seems to be higher in disengaged studyholics than in engaged studyholic, while PS and study-related perfectionism are higher in engaged than in disengaged studyholics. Instead, as far as overstudy climate and worry are concerned, the two kinds of studyholic do not differ. Moreover, for outcomes, disengaged and engaged studyholics do not differ in sleep quality, daytime sleepiness, general stress, and quarrels at university. However, engaged studyholics are more socially impaired (higher family and friends’ complaints and social relationship impairment), which could be linked to their higher time investment in study, while disengaged studyholics are more impaired as concerns their affect and their academic success (lower GPA and higher dropout intention).

### Limitations

One of the limits of this research is related to the sample, which is large, but most of the participants are females. Moreover, another methodological issue concerns the instrument we used for evaluating PS and PC, namely, the Short Almost-Perfect Scale (SAPS) (90). The Italian version (92) does not have fully satisfactory psychometric properties. Hence, the results for perfectionism should be evaluated while taking into account this methodological problem, and we suggest that future studies should analyze the role of PS and PC on studyholism by means of another instrument in order to compare the results with those that we found using the SAPS. Moreover, academic performance is evaluated by means of self-reported GPA; however, this score does not allow differentiating between students that have a high GPA based on many exams and students that have a high GPA that is based only on a few exams. Hence, future research should analyze the effects of studyholism both on GPA and on the total number of exams, as it is possible that studyholics have a high GPA but they are not able to pass all the exams of a year.

### Theoretical and Practical Implications

From a *theoretical* point of view, our results support Loscalzo and Giannini’s (12) conceptualization of studyholism as an internalizing disorder, and more specifically as an obsessive-compulsive-related disorder. Worry, which is a typical internalizing symptom, is the strongest predictor of studyholism. Hence, even though perfectionism (which is another internalizing symptom) does not predict studyholism, the results allow concluding that studyholism seems to be more similar to an obsession than to an addiction toward study.

In addition, the results regarding differences between engaged and disengaged studyholics on outcomes seem to suggest that disengaged studyholics are not the most impaired type [as suggested by Loscalzo and Giannini (12)], as they do not differ in sleep quality impairment, daytime sleepiness, general stress, and quarrels at university. Moreover, disengaged studyholics are more emotionally and academically impaired, as they have more negative affect, lower positive affect and GPAs, and higher dropout intentions. However, engaged studyholics are more socially impaired, as they have higher levels of family and friends’ complaints and social relationship impairment due to study. Hence, these results seem to suggest that both engaged and disengaged studyholics should be considered as clinical forms of overstudying (and not only the disengaged type), while it should be useful to add two specifiers: 1) engagement: high, average, or low; and 2) impairment: academic, social, or academic and social. Also, as suggested by Loscalzo and Giannini (12), it is useful to distinguish between different forms of heavy study investor when analyzing potential studyholism antecedents and outcomes, as the different types of student could have different relationships for the same variables, as shown by this study.

Finally, on the basis of the results that both studyholism and study engagement positively predict higher family and friends’ complaints, we suggest that heavy study investment is not generally a socially acceptable behavior, especially as family and friends’ complaints are even higher for study engagement than for studyholism.

Regarding *preventive and clinical implications*, the results suggest that worry should be the primary focus of interventions aimed at preventing or reducing studyholism; hence, it could be helpful to look for programs that have already been tested for reducing worry, and to apply them in the university context.

Perfectionism and study-related perfectionism do not predict studyholism. However, perfectionism concerns should be addressed as well by interventions aiming at improving students’ wellbeing and academic success, as this variable predicts lower GPA and study engagement, and greater dropout intention and psychological and physical negative outcomes. Hence, reducing perfectionism concerns could help in increasing study engagement and reducing dropout intention. Instead, as far as study-related perfectionism is concerned, we suggest that, even though it is not a predictor of studyholism, it should be addressed by preventive interventions intended to favor students’ wellbeing, as it is a predictor of quarrels with peers and teachers and of family and friends’ complaints and social relationship impairment. However, it is also associated with higher study engagement and GPA; for this reason, students might well believe that it is a positive characteristic that allows them to be successful students. Consequently, interventions should make them aware of the potential social negative outcomes associated with study-related perfectionism and that they may accrue the same positive academic results by means of other behaviors that are not associated with negative outcomes, such as study engagement. In conclusion, preventive interventions that aim to improve students’ wellbeing and academic success (and that do not focus specifically on studyholism) should possibly target worry, as well as PC and study-related perfectionism. In addition, as area of study does not predict studyholism, we suggest that preventive interventions should be spread across all the courses, as each student could potentially be a studyholic, regardless of his/her specific major, and by the first year of college.

Finally, since study engagement predicts social impairment as well as studyholism, we strongly suggest that preventive interventions aiming to favor students’ wellbeing (and not only academic success) should be addressed to engaged students too. The trainings should focus on teaching them how to manage their time in order to have academic success but also leave some time free for friends, family, and leisure time.

In a clinical setting, it is particularly important to distinguish between engaged and disengaged studyholics as they have different relationships with some antecedents and outcomes. More specifically, PC is higher in disengaged studyholics, while PS and study-related perfectionism are higher in engaged studyholics. However, they do not differ in worry and overstudy climate. In addition, while disengaged studyholics are more impaired as far as their affect and their academic success are concerned, engaged studyholics are more socially impaired. Being aware of these differences could help in tailoring the intervention for the specific student.

## Conclusions

This research supports Loscalzo and Giannini’s (12) conceptualization of problematic overstudying as an internalizing disorder, and more specifically as an obsessive-compulsive-related disorder, in contrast with the behavioral addiction model (11). Worry, which is a typical internalizing and OCD symptom, is indeed a strong predictor of studyholism. Hence, the results allow concluding that studyholism seems to be more similar to an obsession than to an addiction toward study.

In addition, the results regarding differences between engaged and disengaged studyholics support Loscalzo and Giannini’s (12) model, which foresee the distinction between these two types of studyholism. However, disengaged studyholics are not the most impaired type [as hypothesized by Loscalzo and Giannini (12)]. Disengaged and engaged studyholics do not differ in sleep quality and general stress; moreover, disengaged studyholics are more emotionally and academically impaired, while engaged studyholics are more socially impaired.

About preventive interventions that aim to improve students’ wellbeing and academic success (and that do not focus specifically on studyholism), they should possibly target worry, as well as PC and study-related perfectionism. In addition, as area of study does not predict studyholism, preventive interventions should be spread across all the courses, as each student could potentially be a studyholic, regardless of his/her specific major, and by the first year of college. Finally, since study engagement predicts social impairment as well as studyholism, we strongly suggest that preventive interventions should be addressed to engaged students too.

Future studies might test if preventive interventions based on these suggestions are effective in reducing studyholism and improving students’ wellbeing and their academic success.

Finally, in a clinical setting, it is particularly important to distinguish between engaged and disengaged studyholics as they have different relationships with some antecedents and outcomes. Being aware of these differences could help in tailoring the intervention for the specific student.

## Ethics Statement

This study was carried out in accordance with the recommendations of the Ethic Committee of the University of Florence and in accordance with the Declaration of Helsinki. The protocol was approved by the Ethic Committee of the University of Florence.

Our study was exempted from written informed consent, which has been obtained instead, as agreed with the Ethical Committee, in the following way: We wrote the usual informed consent information in the first page of the questionnaire. Then, we asked the participants to check a box saying that they agreed to take part in the research by going on and filling out the questionnaire on the following pages.

## Author Contributions

YL developed the study design, conducted the literature review, gathered the data, performed statistical analyses, and wrote the draft of the paper. MG critically revised the statistical analyses and the content of the manuscript.

## Conflict of Interest Statement

The authors declare that the research was conducted in the absence of any commercial or financial relationships that could be construed as a potential conflict of interest.
